# The association between type 2 diabetes and pulmonary cavitation revealed among IGRA-positive tuberculosis patients

**DOI:** 10.3389/fmed.2023.1245316

**Published:** 2023-12-06

**Authors:** Min Yang, Pei Li, Han Liu, Xiaojie Zhu, Guofeng Zhu, Peize Zhang, Guofang Deng

**Affiliations:** ^1^Department of Pulmonary Medicine and Tuberculosis, National Clinical Research Center for Infectious Diseases, Guangdong Provincial Clinical Research Center for Infectious Diseases (Tuberculosis), Shenzhen Clinical Research Center for Tuberculosis, The Third People's Hospital of Shenzhen, Southern University of Science and Technology, Shenzhen, China; ^2^China Institute of Veterinary Drug Control, Beijing, China

**Keywords:** tuberculosis, diabetes mellitus, pulmonary cavitation, interferon-gamma, risk factor

## Abstract

The co-occurrence of tuberculosis (TB) and diabetes mellitus (DM) presents a significant obstacle to TB eradication. Pulmonary cavitation can occur in severe cases of TB, particularly in patients with DM. From 1 May 2014 through 30 June 2019, we conducted a cross-sectional study of 1,658 smear- or culture-confirmed pulmonary TB (PTB) patients at the Second Department of Pulmonary Medicine and Tuberculosis, Shenzhen, China. A total of 861 participants who satisfied the criteria (chest CT scan for cavitation, interferon-gamma release assay (IGRA), diagnosis of diabetes mellitus), with the median age of 36.7 years, 63.6% of male, 79.7% IGRA positive, 13.8% with diabetes, and 40.8% with pulmonary cavitation, were included in the study. The association between diabetes and pulmonary cavitation was confirmed in these TB patients (adjusted OR, 2.54; 95% CI, 1.66–3.94; *p* < 0.001). No associations were observed between diabetes and IGRA, as well as between lung cavitary and IGRA. Based on the criteria of IGRA+/–, pulmonary cavitation+/–, and DM+/–, the further analysis with univariate and multivariate logistic regression were conducted in six subgroups. The significant association between diabetes and pulmonary cavitation was further confirmed in the IGRA+ subgroup (adjusted OR, 3.07; 95% CI, 1.86–5.16; *p* < 0.001) but not observed in IGRA- individuals. This observation suggests that different immunological mechanisms of pulmonary cavitary/DM may be employed in IGRA+ TB patients from IGRA- TB patients.

## 1 Introduction

Tuberculosis (TB) remains a major global health challenge, particularly in low- and middle-income countries. According to the World Health Organization, there were 10.6 million new TB cases and 1.6 million deaths from TB in 2021 (1). China has the world's second largest population of TB patients, with approximately 1 million new TB cases reported each year.

Diabetes, as a metabolic disorder disease, is associated with innate and adaptive immune dysfunctions and alterations in specific cytokines and chemokines (2). Previous studies have reported a paradoxical hyperinflammatory response in TB patients with DM, such as IFN-γ, IL-2, and TNF-a (3–5). There were studies indicating that diabetes mellitus (DM) is associated with an increased risk of TB (6–8). It was reported that individuals with DM have a 2- to 4-fold higher risk of active TB, and up to 30% of individuals with TB are likely to have DM (9).

As a key pathological feature and a dangerous consequence of clinical TB, pulmonary cavitation is associated with poor treatment outcomes, relapse, higher transmission rates, and the development of drug resistance (10). Previous studies proved that the incidence of cavitary TB is higher in diabetic patients compared to non-diabetic patients (11–14). TB individuals with DM experience persistent hyperglycemia, leading to a compilation of aberrant metabolic changes and increased superoxide production, which activates inflammatory pathways and leads to immune system dysfunction, indicating an abnormal and progressive immune response that favors cavitation in diabetic TB individuals (15).

Interferon-γ serves as a crucial lymphokine for the protective immune response to *M. tuberculosis* (16, 17). Reduced sensitivity of IFN-γ release has been demonstrated in humans with physiologically and pathologically immunocompromised factors (18). The interferon-γ release assay (IGRA), measuring early secreted antigenic target 6 (ESAT-6) and culture filtrate protein 10 (CFP-10) to indicate a specific cellular immune response to *M. tuberculosis*, has been routinely used in clinical screening for TB infection. Additionally, it serves as an adjunct diagnostic biomarker for active TB (19). In this study, we assessed whether cavitary or DM is associated with MTB-stimulated interferon-γ secretion in clinical TB patients.

## 2 Materials and methods

### 2.1 Ethics statement

This study was approved by the ethics committee of the Third People's Hospital of Shenzhen (IRB No.: 2021-014-02). All patients' private information was deleted, and each case was coded with a pathology accession number to protect patient privacy. The ethics committee waived the requirement for written informed consent from patients. The study was conducted in accordance with the ethical standards and confidentiality principles outlined in the Helsinki Declaration.

### 2.2 Study population

From 1 May 2014 to 30 June 2019, a total of 1,658 patients with microbiologically confirmed pulmonary TB were recruited in the Second Department of Pulmonary Medicine and Tuberculosis, the Third People's Hospital of Shenzhen. A positive sputum smear or culture was indicative of microbiologically confirmed pulmonary TB, excluding non-mycobacterium pulmonary diseases.

**Figure 1:**
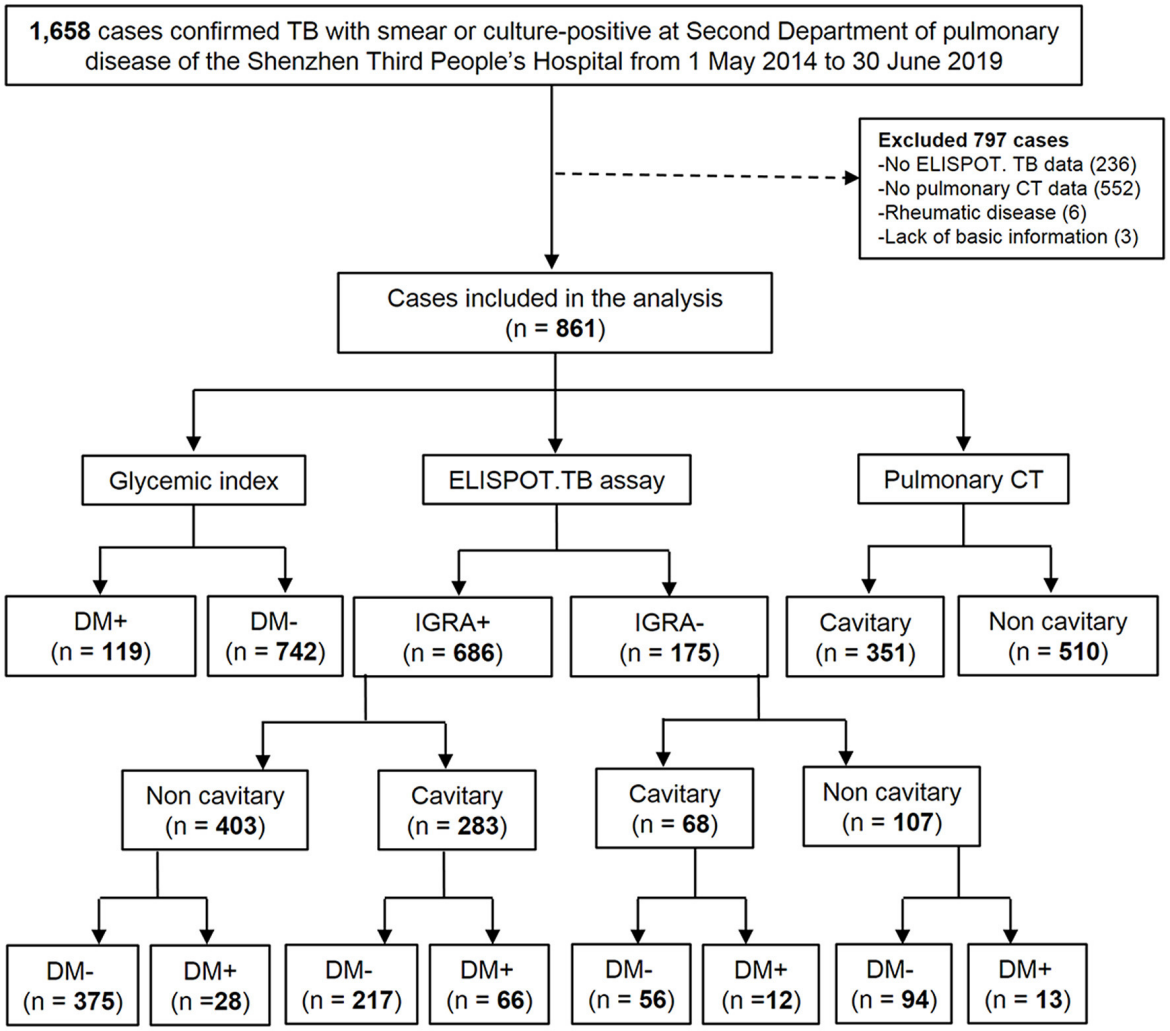
Flow chart of the study population. DM, diabetes mellitus; TB, tuberculosis; CT, thoracic computed tomography; ELISPOT. TB, enzyme-linked immune-spot tuberculosis (ELISPOT. TB) assay (in house). IGRA +, TB patients with positive ELISPOT. TB; IGRA-, TB patients with negative ELISPOT. TB; DM+, TB patients with DM; DM-, TB patients without DM; Cavitary, TB patients with cavitary; Non-cavitary, TB patients without cavitary.

Sociodemographic characteristics, outpatient characteristics, outpatient and inpatient clinical encounters, medication prescriptions and fills, medical conditions, procedures, and laboratory results. Among the 1,658 patients, the exclusion criteria for this study included (1) absence of IGRA results (*n* = 236); (2) lack of CT images (*n* = 552); (3) onset of the rheumatic disease (*n* = 6); and (4) absence of basic information (*n* = 3). Ultimately, 861 patients were enrolled in this study (Figure 1).

### 2.3 Methods

The enzyme-linked immune-spot tuberculosis (ELISPOT. TB) assay was used for screening the interferon-gamma release. Blood glucose and glycated hemoglobin (HbA1c) tests along with chest high-resolution computed tomography (CT) were performed at the patient's first admission. The IGRA assay was performed according to previously published research methods (20) and briefly described as follows: Panel A (peptides of ESAT-6 aa 21 to 40, aa 51 to 70, and aa 71 to 90) and Panel B (peptides of CFP-10 aa 21 to 40, aa 51 to 70, and aa 66 to 85) were used as antigens at a concentration of 10 μg/ml. Peripheral blood mononuclear cells (PBMCs) were freshly isolated from 10 ml of anticoagulated blood samples using the Ficoll density gradient centrifugation method. A total of 2 × 10^5^ cells/well were seeded in duplicate in 96-well plates pre-coated with an anti-IFN-capture monoclonal antibody. The cells were then stimulated with different antigens for 24 h at 37°C, 5% CO_2_. PBMCs in medium alone or stimulated with phytohemagglutinin were used as negative or positive controls. Biotinylated anti-IFN-detection monoclonal antibody was added for 4 h, followed by the addition of streptavidin-alkaline phosphatase conjugate for 1 h. Subsequently, nitroblue tetrazolium-BCIP (5-bromo-4-chloro-3-indolylphosphate) chromogenic substrate was added for staining. The spots were counted using BioReader 4,000 Pro-X (Biosys, Germany). The test result of ELISPOT.TB assay was considered positive if either or both of Panel A had 18 or Panel B had 11 or more spots than the negative control.

Diabetes mellitus was defined according to the diagnostic criteria established by the WHO, and patients with HbA1c ≥ 6.5% were commonly diagnosed with DM (21). The baseline information on demographic variables (for e.g., sex, age, ethnicity, marital status, and work status), behavioral risk factors (for e.g., smoking and alcohol use) and paraclinical data [for e.g., hemoglobin (HGB)] along with serum albumin (ALB), white blood cell count (WBC), lymphocyte count (LYN), neutrophilic granulocyte count (GRA), monocyte count (MONO), and C-reactive protein (CRP) were considered as confounders. The CT scanners used in this study included CT64 (China), Light Speed 16 and General Electric (GE) Revolution CT256 from United Imaging. The machine (Toshiba Asteion; Toshiba, Tokyo, Japan) parameters were as follows: 1.15-mm section thickness, 3-mm gap, 1- or 2-s scanning time per section, 120 kV, and 200 mA. The images were photographed at the lungs (window width, 1,800 HU, window level, 400 HU). Two experienced radiologists reviewed the CT images and reached a consensus decision at the Third People's Hospital of Shenzhen. The definition of pulmonary cavitation can be referred in this article (3), and a representative CT image is displayed in Supplementary Figure 1.

### 2.4 Statistical analysis

The study data were entered into a spreadsheet and analyzed using the statistical software R (version 4.1.2) (22). Bioinformatic analysis was performed using the OmicStudio tools at https://www.omicstudio.cn.

Bivariate associations were analyzed using chi-square tests for categorical variables and the Wilcoxon rank sum or Kruskal–Wallis tests for continuous variables. A two-sided *p*-value < 0.05 was considered statistically significant. To identify the risk factors associated with the presence of pulmonary cavitation in TB patients, univariate and multivariate logistic regression models were developed. The baseline information on demographic variables (for e.g., sex, age, ethnicity, marital status, and work status), behavioral risk factors (for e.g., smoking and alcohol use), and paraclinical data (for e.g., HGB, ALB, WBC, LYN, GRA, MONO, and CRP) were considered as confounders. The odds ratios (ORs) and their 95% confidence intervals (95% CIs) were also calculated to estimate the degree of association between different variables and pulmonary cavitation. Variables with a *p*-value ≤ 0.20 on the univariate analysis were initially offered to a saturated multivariate logistic regression model. The optimized model was generated using a “both” stepwise process, and the most appropriate model was determined by assessing the likelihood test and the minimum Akaike information criterion (AIC) value (23). The variance inflation factor (VIF) was used to detect multicollinearity in the regression analysis. A variable with VIF >5 was considered to have multicollinearity, and variables with multicollinearity were manually removed step by step until it was eliminated. The model was further assessed using the Hosmer–Lemeshow goodness-of-fit test. A receiver operating characteristic (ROC) curve was produced to display the predictive accuracy of the model using the R package “pROC.” The “lme4” and “gtsummary” packages were used to build and display the univariate and multivariate logistic regression models (24). The “tidyverse” package was used for data import, manipulation, and visualization (25). Additionally, we adopted a similar approach as above to explore the potential association of predictors with IGRA and the relationship between predictors and pulmonary cavitation depending on the IGRA results.

## 3 Results

### 3.1 Study population and clinical characteristics

The study sample comprised 861 microbiologically confirmed pulmonary TB patients. The mean (SD) age of the patients was 36.7 (13.0) years, with 548 (63.6%) being male. Among them, 351 (40.8%) were diagnosed with cavitary PTB as determined by CT findings, and 119 (13.8%) had diabetes mellitus (DM+). Additionally, 686 (79.7%) were tested positive ELISPOT.TB (IGRA+). The baseline characteristics, include marital status, work, ethnicity, smoking and alcohol use, white blood cell count, neutrophilic granulocyte count, lymphocyte count, monocyte count, hemoglobin, albumin, and C-reactive protein, were shown in Supplementary Table 1.

### 3.2 Univariate and multivariate logistic regression analyses of risk factors for cavitary, DM, and IGRA in TB patients

We first tested the risk factors for the lung cavitation. The analysis data in Table 1 suggest that gender (adjusted OR, 0.65; 95% CI, 10.46–0.92; *p* = 0.016), work status (adjusted OR, 20.60; 95% CI, 0.44–0.82; *p* = 0.001), ethnicity (adjusted OR, 3.00; 95% CI, 1.92–7.38; *p* = 0.013), recent alcohol use (adjusted OR, 1.79; 95% CI, 1.10–2.94; *p* = 0.019), and smoking cigarettes (adjusted OR, 1.58; 95% CI, 1.05–2.40; *p* = 0.030) were significantly associated with pulmonary cavitation. Among the clinical characteristics, diabetes and WBC count (adjusted OR, 1.10; 95% CI, 1.05–1.16; *p* < 0.001) were significant risk factors for pulmonary cavitation. The significant association between cavitary and diabetes was identified in the TB group (adjusted OR, 2.54; 95% CI, 1.66–3.94; *p* < 0.001). Univariate and multivariate logistic regression analyses found no statistical significant differences between cavitary and IGRA in these TB patients (OR, 1.10; 95% CI, 0.79-1.56; p= 0.60).

**Table 1:** Univariate and multivariate logistic regression analyses of risk factors for pulmonary cavitation in TB patients (*n* = 861).

	**Statistics**		**Univariate analysis**			**Multivariable analysis**		
**Characteristic**	**Non-cavitary**, ***N*** = **510**^1^	**Cavitary, N** = **351**^1^	**Crude OR** ^2^	**95% CI** ^2^	***p*** **-value**	**Adjusted OR** ^2^	**95% CI** ^2^	***p*** **-value**
**Gender**								
Male	285 (55.9%)	263 (74.9%)	Reference			Reference		
Female	225 (44.1%)	88 (25.1%)	0.42	0.31, 0.57	< 0.001	0.65	0.46, 0.92	**0.016**
Age	35.70 (12.7)	38.10 (13.3)	1.01	1.00, 1.02	0.010			
**Marital status**								
Single	191 (37.5%)	120 (34.2%)	Reference			Reference		
Married	319 (62.5%)	231 (65.8%)	1.15	0.87, 1.53	0.30			
**Work**								
Unemployed	140 (27.5%)	130 (37.0%)	Reference			Reference		
Employed	370 (72.5%)	221 (63.0%)	0.64	0.48, 0.86	0.003	0.60	0.44, 0.82	**0.001**
**Ethnicity**								
Han	501 (98.2%)	334 (95.2%)	Reference			Reference		
Others	9 (1.8%)	17 (4.8%)	2.83	1.28, 6.72	0.013	**3.00**	1.29, 7.38	**0.013**
**Smokers**								
Never	400 (78.4%)	201 (57.3%)	Reference			Reference		
Current	87 (17.1%)	121 (34.5%)	2.77	2.01, 3.83	< 0.001	**1.58**	1.05, 2.40	**0.030**
Former	23 (4.5%)	29 (8.3%)	2.51	1.42, 4.49	0.002	1.42	0.74, 2.73	0.30
**Drinkers**								
Never	462 (90.6%)	272 (77.5%)	Reference			Reference		
Current	41 (8.0%)	75 (21.4%)	3.11	2.07, 4.71	< 0.001	1.79	1.10, 2.94	**0.019**
Former	7 (1.4%)	4 (1.1%)	0.97	0.25, 3.24	>0.90	0.39	0.10, 1.39	0.20
**IGRA**								
Negative	107 (21.0%)	68 (19.4%)	Reference			Reference		
Positive	403 (79.0%)	283 (80.6%)	1.10	0.79, 1.56	0.60			
**Diabetes**								
No	469 (92.0%)	273 (77.8%)	Reference			Reference		
Yes	41 (8.0%)	78 (22.2%)	3.27	2.19, 4.94	< 0.001	**2.54**	1.66, 3.94	**< 0.001**
WBC	7.2 (2.7)	8.4 (3.2)	1.14	1.09, 1.20	< 0.001	**1.10**	1.05, 1.16	**< 0.001**
GRA	5.1 (3.7)	6.1 (2.9)	1.11	1.06, 1.17	< 0.001			
LYN	1.4 (0.6)	1.4 (0.6)	0.99	0.80, 1.23	>0.90			
MONO	0.7 (0.7)	0.7 (0.3)	1.05	0.83, 1.35	0.60			
HGB	123.4 (20.6)	122.2 (20.6)	1.00	0.99, 1.00	0.40			

1 n (%); Mean (SD); Reference used as control for comparison.

2 OR = Odds Ratio, CI = Confidence Interval. WBC, white blood cell; GRA, neutrophilic granulocyte; LYN, lymphocyte; MONO, monocyte; HGB, hemoglobin; ALB, albumin; and CRP, C-reactive protein. Significant differences (*p* < 0.05) are bolded.

We then tested the risk factors for diabetes mellitus. As shown in Table 2, the gender (adjusted OR, 0.43; 95% CI, 0.24–0.72; *p* = 0.002), age (adjusted OR, 1.08; 95% CI, 1.06–1.10; *p* < 0.001), marital status (adjusted OR, 3.87; 95% CI, 1.75–9.81; *p* = 0.002), and WBC count (adjusted OR, 1.10; 95% CI, 1.02–1.17; *p* < 0.001) were significantly associated with diabetes. Furthermore, no statistical significant difference between diabetes and IGRA was found (OR, 0.95; 95% CI, 0.60–1.56; *p* = 0.842).

**Table 2:** Univariate and multivariate logistic regression analyses of risk factors for type 2 diabetes in TB patients (*n* = 861).

	**Statistics**		**Univariate analysis**			**Multivariable analysis**		
**Characteristic**	**Diabetes**, ***N** =* **119**^1^	**Non-diabetes**, ***N** =* **742**^1^	**Crude OR** ^2^	**95% CI** ^2^	***p*** **-value**	**Adjusted OR** ^2^	**95% CI** ^2^	***p*** **-value**
**Gender**								
Male	98 (82.4%)	450 (74.9%)	Reference			Reference		
Female	21 (17.6%)	292 (25.1%)	0.33	0.20, 0.53	< 0.001	0.43	0.24, 0.72	**0.002**
Age	49.6 (9.2)	34.6 (12.3)	1.10	1.08, 1.12	< 0.001	1.08	1.06, 1.10	**< 0.001**
**Marital status**								
Single	7 (5.9%)	304 (41.0%)	Reference			Reference		
Married	112 (94.1%)	438 (59.0%)	11.10	5.48, 26.59	< 0.001	3.87	1.75, 9.81	**0.002**
**Work**								
Unemployed	77 (64.7%)	514 (69.3%)	Reference					
Employed	42 (35.3%)	228 (30.7%)	1.23	0.81, 1.84	0.319			
**Ethnicity**								
Han	117(98.3%)	718 (96.8%)	Reference					
Others	2 (1.7%)	24 (3.2%)	0.51	0.08, 1.75	0.366			
**Smokers**								
Never	56 (47.1%)	545 (73.5%)	Reference					
Current	48(40.3%)	160 (21.6%)	2.92	1.91, 4.46	< 0.001			
Former	15 (12.6%)	37 (5.0%)	3.94	1.99, 7.52	< 0.001			
**Drinkers**								
Never	88 (73.9%)	646 (87.1%)	Reference					
Current	27 (22.7%)	89 (12.0%)	2.23	1.35, 3.58	0.001			
Former	4 (3.4%)	7 (0.9%)	4.19	1.08, 14.18	0.024			
**IGRA**								
Negative	25 (21.0%)	150 (20.2%)	Reference					
Positive	94 (79.0%)	592 (79.8%)	0.95	0.60, 1.56	0.842			
**Cavitation**								
Non-cavitary	78 (65.5%)	273 (36.8%)	Reference					
Cavitary	41 (34.5%)	469 (63.2%)	3.27	2.19, 4.94	< 0.001			
WBC	8.6 (3.1)	7.5 (2.9)	1.11	1.05, 1.18	< 0.001	**1.10**	1.02, 1.17	**< 0.001**
GRA	6.3(3.0)	5.4 (3.5)	1.06	1.01, 1.12	0.031			
LYN	1.4 (0.6)	1.4 (0.6)	1.14	0.84, 1.52	0.389			
MONO	0.7 (0.3)	0.7 (0.6)	1.08	0.77, 1.39	0.554			
HGB	125.6 (19.7)	122.5 (20.7)	1.01	1.00, 1.02	0.125			

1 n (%); Mean (SD); Reference used as control for comparison.

2 OR = Odds Ratio, CI = Confidence Interval. WBC, white blood cell; GRA, neutrophilic granulocyte; LYN, lymphocyte; MONO, monocyte; HGB, hemoglobin; ALB, albumin; and CRP, C-reactive protein. Significant differences (*p* < 0.05) are bolded.

We also tested the risk factors for the IGRA and the results are presented in Table 3. It can be observed that age is a significant risk factor (adjusted OR, 0.98; 95% CI, 0.97–1.00; *p* = 0.008). Moreover, significant associations between IGRA and two clinical characteristics were observed: one was WBC (adjusted OR, 0.92; 95% CI, 0.86–0.98; *p* = 0.008) and another one was HGB (adjusted OR, 1.01; 95% CI, 1.00–1.02; *p* = 0.046). No statistically significant difference between IGRA and diabetes was found (OR, 0.95; 95% CI, 0.60–1.56; *p* = 0.80), nor was there a significant difference between IGRA and cavitary findings (OR, 1.10; 95% CI, 0.79–1.56; *p* = 0.60) in these TB patients.

**Table 3:** Univariate and multivariate logistic regression analyses of risk factors for IGRA in TB patients (*n* = 861).

	**Statistics**		**Univariate analysis**			**Multivariable analysis**		
**Characteristic**	**IGRA (-)**, ***N** =* **175**^1^	**IGRA (**+**)**, ***N** =* **686**^1^	**Crude OR** ^2^	**95% CI** ^2^	***p*** **-value**	**Adjusted OR** ^2^	**95% CI** ^2^	***p*** **-value**
**Gender**								
Male	110 (62.9%)	438 (63.8%)	Reference			Reference		
Female	65 (37.1%)	248 (36.2%)	0.96	0.68, 1.36	0.8			
Age	39.4 (13.8)	36.0 (12.7)	0.98	0.97, 0.99	0.002	0.98	0.97, 1.00	**0.008**
**Marital status**								
Single	53 (30.3%)	258 (37.6%)	Reference			Reference		
Married	122 (69.7%)	428 (62.4%)	0.72	0.50, 1.03	0.073			
**Work**								
Unemployed	53 (30.3%)	217 (31.6%)	Reference			Reference		
Employed	122 (69.7%)	469 (68.4%)	0.94	0.65, 1.34	0.70			
**Ethnicity**								
Han	170 (97.1%)	665 (96.9%)	Reference			Reference		
Others	5 (2.9%)	21 (3.1%)	1.07	0.43, 3.25	0.90			
**Smokers**								
Never	123 (70.3%)	478 (69.7%)	Reference			Reference		
Current	40 (22.9%)	168 (24.5%)	1.08	0.73, 1.62	0.70			
Former	12 (6.9%)	40 (5.8%)	0.86	0.45, 1.75	0.70			
**Drinkers**								
Never	151 (86.3%)	583 (85.0%)	Reference			Reference		
Current	20 (11.4%)	96 (14.0%)	1.24	0.76, 2.13	0.40			
Former	4 (2.3%)	7 (1.0%)	0.45	0.14, 1.75	0.20			
**Diabetes**								
No	150 (85.7%)	592 (86.3%)	Reference			Reference		
Yes	25 (14.3%)	94 (13.7%)	0.95	0.60, 1.56	0.80			
**Cavitation**								
Non-cavitary	107 (61.1%)	403 (58.7%)	Reference			Reference		
Cavitary	68 (38.9%)	283 (41.3%)	1.10	0.79, 1.56	0.60			
WBC	8.0 (3.2)	7.6 (2.9)	0.96	0.91, 1.01	0.10	**0.92**	0.86, 0.98	**0.008**
GRA	5.9 (3.1)	5.4 (3.5)	0.96	0.92, 1.01	0.095			
LYN	1.3 (0.7)	1.4 (0.6)	1.44	1.09, 1.93	0.013	1.25	0.92, 1.72	0.20
MONO	0.6 (0.3)	0.7 (0.7)	1.49	0.96, 2.60	0.14	1.91	1.07, 3.75	0.053
HGB	118.7 (21.9)	124.0 (20.1)	1.01	1.00, 1.02	0.003	**1.01**	1.00, 1.02	**0.046**

1 *n* (%); Mean (SD); Reference used as control for comparison.

2 OR, Odds Ratio; CI, Confidence Interval. WBC, white blood cell; GRA, neutrophilic granulocyte; LYN, lymphocyte; MONO, monocyte; HGB, hemoglobin; ALB, albumin; and CRP, C-reactive protein. Significant differences (*p* < 0.05) are bolded.

### 3.3 Univariate and multivariate logistic regression analyses of risk factors in the specific subgroup of TB patients

According to the pulmonary cavitation+/–, diabetes+/–, and IGRA+/–, we categorized the enrolled TB patients into six specific subgroups and further conducted the risk factors analysis.

A total of 351 TB patients were categorized as the subgroup cavitary+ based on the presence of cavities in their lungs. No significant risk factors of IGRA were observed (Supplementary Table 2a), and no significant association between IGRA and diabetes (OR, 1.42; 95% CI, 0.74-2.92; *p* = 0.314) was established. As shown in Supplementary Table 2b, age (adjusted OR, 1.08; 95% CI, 1.05–1.11; *p* < 0.001), marital status (adjusted OR, 6.18; 95% CI, 2.03–26.86; *p* = 0.004), and HGB (adjusted OR, 1.02; 95% CI, 1.00–1.03; *p* = 0.019) were risk factors for diabetes. Similarly, there was no statistical significant difference between IGRA and diabetes (OR, 1.42; 95% CI, 0.74–2.92; *p* = 0.314) in the cavitary+ subgroup.

In the subgroup cavitary- (*n* = 510), age (adjusted OR, 0.97; 95% CI, 0.96–0.99; *p* = 0.0008), WBC (adjusted OR, 0.9; 95% CI, 0.84–0.97; *p* = 0.0077), and HGB (adjusted OR, 1.01; 95% CI, 1.0–1.02; *p* = 0.0496) were observed as significant risk factors of IGRA. There was no significant association between IGRA and diabetes (OR, 0.54; 95% CI, 0.27–1.11; *p* = 0.0824) in this subgroup (Supplementary Table 3a). The significant association between diabetes, age (adjusted OR, 1.10; 95% CI, 1.07–1.14; *p* < 0.001), and gender (adjusted OR, 0.38; 95% CI, 0.15–10.86; *p* = 0.027) were observed in cavitary- TB patients (Supplementary Table 3b).

In the subgroup diabetes+ (*n* = 119), there was no risk factors of IGRA were observed (Supplementary Table 4a). Several risk factors for cavitary, such as gender (adjusted OR, 0.54; 95% CI, 0.38–0.77; *p* < 0.001), recent alcohol use (adjusted OR, 2.26; 95% CI, 1.39–3.70; *p* = 0.001), work status (adjusted OR, 1.61; 95% CI, 1.14–2.26; *p* = 0.006), ethnicity (adjusted OR, 3.50; 95% CI, 1.46–9.00; *p* = 0.006), and WBC (adjusted OR, 1.12; 95% CI, 1.06–1.19; *p* < 0.001) were observed in this subgroup (Supplementary Table 4b). There was no significant association between IGRA and cavitary (adjusted OR, 2.1; 95% CI, 0.80–5.52; *p* = 0.129) in the diabetes+ subgroup.

In the subgroup diabetes- (*n* = 742), age (adjusted OR, 0.98; 95% CI, 0.96–0.99; *p* = 0.001) and HGB (adjusted OR, 1.01; 95% CI, 1.00–1.02; *p* = 0.017) were observed as significant risk factors of IGRA. There was no significant association between IGRA and diabetes (OR, 0.97; 95% CI, 0.67–1.41; *p* = 0.878) in this subgroup (Supplementary Table 5a). No risk factor was observed for the diabetes in these TB patients (Supplementary Table 5b).

In the subgroup IGRA+ (*n* = 686), work (adjusted OR, 0.60; 95% CI, 0.42–0.86; *p* = 0.005), ethnicity (adjusted OR, 4.12; 95% CI, 1.56–12.2; *p* = 0.006), smoking (adjusted OR, 1.65; 95% CI, 1.03–2.66; *p* = 0.037), drinking (adjusted OR, 1.85; 95% CI, 1.06–13.26; *p* = 0.031), WBC (adjusted OR, 1.14; 95% CI, 1.07–1.22; *p* < 0.001), and diabetes (adjusted OR, 3.07; 95% CI, 1.86–5.16; *p* < 0.001) were observed as significant risk factors of cavitary (Table 4). As shown in Table 5, gender (adjusted OR, 0.41; 95% CI, 0.21–0.78; *p* = 0.009), age (adjusted OR, 1.08; 95% CI, 1.06–1.11; *p* < 0.001), marital status (adjusted OR, 3.76; 95% CI, 1.57–10.45; *p* = 0.005), and cavitary (adjusted OR, 3.07; 95% CI, 1.86–5.16; *p* < 0.001) were the risk factors for diabetes.

**Table 4:** Univariate and multivariate logistic regression analyses of risk factors for pulmonary cavitation in TB patients who tested IGRA positive (*n* = 686).

	**Statistics**		**Univariate analysis**			**Multivariable analysis**		
**Characteristic**	**Non-cavitary**, ***N** =* **403**^1^	**Cavitary**, ***N** =* **283**^1^	**Crude OR** ^2^	**95% CI** ^2^	***p*** **-value**	**Adjusted OR** ^2^	**95% CI** ^2^	***p*** **-value**
**Gender**								
Male	224 (55.6%)	214 (75.6%)	Reference			Reference		
Female	179 (44.4%)	69 (24.4%)	0.40	0.29, 0.56	< 0.001	0.68	0.46, 1.02	0.064
Age	34.7 (12.1)	37.9 (13.3)	1.02	1.01, 1.03	0.001			
**Marital**								
Single	161 (40.0%)	97 (34.3%)	Reference			Reference		
Married	242 (60.0%)	186 (65.7%)	1.28	0.93, 1.75	0.13			
**Work**								
Unemployed	111 (27.5%)	106 (37.5%)	Reference			Reference		
Employed	292 (72.5%)	177 (62.5%)	0.63	0.46, 0.88	0.006	0.60	0.42, 0.86	**0.005**
**Ethnicity**								
Han	397 (98.5%)	268 (94.7%)	Reference			Reference		
Others	6 (1.5%)	15 (5.3%)	3.70	1.48, 10.5	0.007	4.12	1.56, 12.2	**0.006**
**Smokers**								
Never	319 (79.2%)	159 (56.2%)	Reference			Reference		
Current	68 (16.9%)	100 (35.3%)	2.95	2.06, 4.25	< 0.001	1.65	1.03, 2.66	**0.037**
Former	16 (4.0%)	24 (8.5%)	3.01	1.57, 5.93	0.001	1.52	0.71, 3.28	0.30
**Drinkers**								
Never	367 (91.1%)	216 (76.3%)	Reference			Reference		
Current	32 (7.9%)	64 (22.6%)	3.40	2.17, 5.42	< 0.001	1.85	1.06, 3.26	**0.031**
Former	4 (1.0%)	3 (1.1%)	1.27	0.25, 5.83	0.80	0.56	0.10, 2.78	0.50
**Diabetes**								
No	375 (93.1%)	217 (76.7%)	Reference			Reference		
Yes	28 (6.9%)	66 (23.3%)	4.07	2.57, 6.62	< 0.001	**3.07**	1.86, 5.16	**< 0.001**
WBC	7.0 (2.4)	8.4 (3.3)	1.19	1.13, 1.27	< 0.001	**1.14**	1.07, 1.22	**< 0.001**
GRA	4.9 (3.7)	6.1 (3.1)	1.15	1.08, 1.22	< 0.001			
LYN	1.4 (0.6)	1.4 (0.6)	1.03	0.81, 1.32	0.80			
MONO	0.7 (0.8)	0.7 (0.3)	1.05	0.82, 1.36	0.70			
HGB	124.5 (20.1)	123.4 (20.1)	1.00	0.99, 1.00	0.50			

1 *n* (%); Mean (SD); Reference used as control for comparison.

2 OR, Odds Ratio; CI, Confidence Interval. WBC, white blood cell; GRA, neutrophilic granulocyte; LYN, lymphocyte; MONO, monocyte; HGB, hemoglobin; ALB, albumin; and CRP, C-reactive protein. Significant differences (*p* < 0.05) are bolded.

**Table 5:** Univariate and multivariate logistic regression analyses of risk factors for type 2 diabetes in TB patients who tested IGRA positive (*n* = 686).

	**Statistics**		**Univariate analysis**			**Multivariable analysis**		
**Characteristic**	**Diabetes, (*****N** =* **94)**^1^	**Non-diabetes, (*****N** =* **592)**^1^	**Crude OR** ^2^	**95% CI** ^2^	***p*** **-value**	**Adjusted OR** ^2^	**95% CI** ^2^	***p*** **-value**
**Gender**								
Male	80 (85.1%)	358 (60.5%)	Reference			Reference		
Female	14(14.9%)	234 (39.5%)	0.27	0.14, 0.47	< 0.001	0.41	0.21, 0.78	**0.009**
Age	49.5 (9.4)	33.8 (11.8)	1.10	1.08, 1.13	< 0.001	1.08	1.06, 1.11	**< 0.001**
**Marital status**								
Single	6 (6.4%)	252 (42.6%)	Reference			Reference		
Married	88 (93.6%)	340 (57.4%)	10.87	5.08, 28.25	< 0.001	3.76	1.57, 10.45	**0.005**
**Work**								
Unemployed	58 (61.7%)	411 (69.4%)	Reference					
Employed	36 (38.3%)	181 (30.6%)	1.41	0.89, 2.20	0.136			
**Ethnicity**								
Han	92(97.9%)	573 (96.8%)	Reference					
Others	2 (2.1%)	19 (3.2%)	0.66	0.10, 2.31	0.574			
**Smokers**								
Never	43 (45.7%)	435 (73.5%)	Reference					
Current	39 (41.5%)	129 (21.8%)	3.06	1.90, 4.92	< 0.001			
Former	12 (12.8%)	28 (4.7%)	4.34	2.00, 8.98	< 0.001			
**Drinkers**								
Never	67 (71.3%)	516 (87.2%)	Reference					
Current	23 (24.5%)	73 (12.3%)	2.42	1.40, 4.09	0.001			
Former	4 (4.3%)	3 (0.5%)	10.27	2.22, 53.07	0.002			
**Cavitation**								
Non-cavitary	66 (70.2%)	217 (36.7%)	Reference			Reference		
Cavitary	28 (29.8%)	375 (63.3%)	4.07	2.57, 6.62	< 0.001	**3.07**	1.86, 5.16	**< 0.001**
WBC	8.5 (2.9)	7.5 (2.8)	1.11	1.03, 1.19	0.002	1.07	0.99, 1.16	0.102
GRA	6.0 (2.7)	5.3 (3.6)	1.04	0.99, 1.11	0.102			
LYN	1.5 (0.6)	1.4 (0.6)	1.27	0.90, 1.75	0.155			
MONO	0.8 (0.3)	0.7 (0.7)	1.09	0.77, 1.40	0.533			
HGB	127.3 (19.1)	123.5 (20.2)	1.01	1.00, 1.02	0.091			

1 *n* (%); Mean (SD); Reference used as control for comparison.

2 OR, Odds Ratio; CI, Confidence Interval. WBC, white blood cell; GRA, neutrophilic granulocyte; LYN, lymphocyte; MONO, monocyte; HGB, hemoglobin; ALB, albumin; and CRP, C-reactive protein. Significant differences (*p* < 0.05) are bolded.

In the subgroup IGRA- (*n* = 175), gender was observed as the only significant risk factor of cavitary (adjusted OR, 0.51; 95% CI, 0.26–0.98; *p* = 0.046) (Table 6). And only the age (adjusted OR, 1.06; 95% CI, 1.01–1.10; *p* = 0.011) was observed as the risk factor of diabetes in this subgroup (Table 7).

**Table 6:** Univariate and multivariate logistic regression analyses of risk factors for pulmonary cavitation in TB patients who tested IGRA negative (*n* = 175).

	**Statistics**		**Univariate analysis**			**Multivariable analysis**		
**Characteristic**	**Non-cavitary**, ***N** =* **107**^1^	**Cavitary**, ***N** =* **68**^1^	**Crude OR** ^2^	**95% CI** ^2^	***p*** **-value**	**Adjusted OR** ^2^	**95% CI** ^2^	***p*** **-value**
**Gender**								
Male	61 (57.0%)	49 (72.1%)	Reference			Reference		
Female	46 (43.0%)	19 (27.9%)	0.51	0.26, 0.98	0.046	0.51	0.26, 0.98	**0.046**
Age	39.7 (14.1)	38.9 (13.5)	1.00	0.97, 1.02	0.70			
**Marital**								
Single	30 (28.0%)	23 (33.8%)	Reference			Reference		
Married	77 (72.0%)	45 (66.2%)	0.76	0.40, 1.48	0.40			
**Work**								
Unemployed	29 (27.1%)	24 (35.3%)	Reference			Reference		
Employed	78 (72.9%)	44 (64.7%)	0.68	0.35, 1.32	0.30			
**Ethnicity**								
Han	104 (97.2%)	66 (97.1%)	Reference			Reference		
Others	3 (2.8%)	2 (2.9%)	1.05	0.14, 6.50	>0.90			
**Smokers**								
Never	81 (75.7%)	42 (61.8%)	Reference			Reference		
Current	19 (17.8%)	21 (30.9%)	2.13	1.03, 4.43	0.040			
Former	7 (6.5%)	5 (7.4%)	1.38	0.39, 4.58	0.60			
**Drinkers**								
Never	95 (88.8%)	56 (82.4%)	Reference			Reference		
Current	9 (8.4%)	11 (16.2%)	2.07	0.81, 5.44	0.13			
Former	3 (2.8%)	1 (1.5%)	0.57	0.03, 4.54	0.60			
**Diabetes**								
No	94 (87.9%)	56 (82.4%)	Reference			Reference		
Yes	13 (12.1%)	12 (17.6%)	1.55	0.65, 3.65	0.30			
WBC	7.9 (3.6)	8.2 (2.6)	1.02	0.93, 1.12	0.60			
GRA	5.8 (3.5)	6.1 (2.4)	1.04	0.94, 1.14	0.50			
LYN	1.3 (0.7)	1.2 (0.6)	0.83	0.52, 1.31	0.40			
MONO	0.6 (0.3)	0.6 (0.3)	1.04	0.40, 2.64	>0.90			
HGB	119.5 (22.0)	117.5 (21.9)	1.00	0.98, 1.01	0.60			

1 *n* (%); Mean (SD); Reference used as control for comparison.

2 OR, Odds Ratio; CI, Confidence Interval. WBC, white blood cell; GRA, neutrophilic granulocyte; LYN, lymphocyte; MONO, monocyte; HGB, hemoglobin; ALB, albumin; and CRP, C-reactive protein. Significant differences (*p* < 0.05) are bolded.

**Table 7:** Univariate and multivariate logistic regression analyses of risk factors for type 2 diabetes in TB patients who tested IGRA negative (*n* = 175).

	**Statistics**		**Univariate analysis**			**Multivariable analysis**		
**Characteristic**	**Diabetes, (*****N** =* **25)**^1^	**Non-diabetes, (*****N** =* **150)**^1^	**Crude OR** ^2^	**95% CI** ^2^	***p*** **-value**	**Adjusted OR** ^2^	**95% CI** ^2^	***p*** **-value**
**Gender**								
Male	18 (72.0%)	92 (61.3%)	Reference					
Female	7 (28.0%)	58 (38.7%)	0.62	0.23, 1.51	0.310			
Age	50.2 (8.4)	37.6 (13.8)	1.08	1.04, 1.12	< 0.001	1.06	1.01, 1.10	**0.011**
**Marital**								
Single	1 (4.0%)	52 (34.7%)	Reference			Reference		
Married	24 (96.0%)	98 (65.3%)	12.73	2.58, 230.83	< 0.001	4.57	0.73, 90.01	0.173
**Work**								
Unemployed	19 (76.0%)	103 (68.7%)	Reference					
Employed	6 (24.0%)	47 (31.3%)	0.69	0.24, 1.76	0.462			
**Ethnicity**								
Han	25 (100.0%)	145 (96.7%)	Reference					
Others	0 (0%)	5 (3.3%)	-^NA^	-^NA^	-^NA^			
**Smokers**								
Never	13 (52.0%)	110 (73.3%)	Reference					
Current	9 (36.0%)	31 (20.7%)	2.46	0.94, 6.24	0.061			
Former	3 (12.0%)	9 (6.0%)	2.82	0.57, 10.92	0.154			
**Drinkers**								
Never	21 (84.0%)	130 (86.7%)	Reference					
Current	4 (16.0%)	16 (10.7%)	1.55	0.41, 4.72	0.471			
Former	0 (0%)	4 (2.7%)	-^NA^	-^NA^	-^NA^			
**Cavitation**								
Non-cavitary	12 (48.0%)	56 (37.3%)	Reference					
Cavitary	13 (52.0%)	94 (62.7%)	1.55	0.65, 3.65	0.313			
WBC	9.1 (3.7)	7.8 (3.1)	1.10	0.98, 1.24	0.085			
GRA	7.1 (3.7)	5.7 (2.9)	1.13	1.00, 1.28	0.039	1.12	0.98, 1.29	0.086
LYN	1.2 (0.6)	1.3 (0.7)	0.77	0.38, 1.47	0.451			
MONO	0.6 (0.3)	0.6 (0.3)	0.99	0.25, 3.49	0.985			
HGB	119.4 (20.9)	118.6 (22.2)	1.00	0.98, 1.02	0.862			

1 n (%); Mean (SD); Reference used as control for comparison; NA, not available.

2 OR, Odds Ratio; CI, Confidence Interval. WBC, white blood cell; GRA, neutrophilic granulocyte; LYN, lymphocyte; MONO, monocyte; HGB, hemoglobin; ALB, albumin; and CRP, C-reactive protein. Significant differences (*p* < 0.05) are bolded.

## 4 Discussion

The univariate and multivariate logistic regression models have been established for data analysis in this project. As shown in Table 2, diabetes was significantly associated with pulmonary cavitation in the univariate analysis (OR, 3.27; 95% CI, 2.19–4.94; *p* < 0.001), and this association was not significant in the multivariate regression analysis. Additionally, in Table 1, the significant association between cavitary and diabetes was identified in the TB group (adjusted OR, 2.54; 95% CI, 1.66–3.94; *p* < 0.001). An ROC curve was generated to assess the predictive value of the constructed model. The model exhibited intermediate accuracy with an area under the ROC curve (AUC) of 0.706, indicating the high accuracy of this model. In this smear or culture-positive, clinical, and 5-year population-based cross-sectional study, the investigation of the association between clinical characteristics and pulmonary cavitation among TB patients was conducted. We found consistent evidence for an increased risk of cavitary TB with diabetes. Additionally, the significant association between diabetes and cavitary has been narrowed to “IGRA positive” TB patients rather than “IGRA negative” TB patients.

Hyperglycemia significantly affected the presentation of radiographic manifestations and was associated with severe TB (26). The possible underlying mechanism was considered to be an impaired immune function in patients with diabetes. IFN-γ, TNF-α, IL-17, and IL-23 play crucial roles in the induction and maintenance of protective immune responses against TB (27). Accumulating evidence has shown that IFN-γ is responsible for driving cell-mediated immune responses through production by Th1 cells and has regulatory properties by acting as an inducer of Th2 responses. Using chronic M.tb infection in hyperglycemic mice, adaptive immunity was found to be delayed, as shown by reduced early production of IFN-γ in the lungs and by the presence of fewer M.tb antigen (ESAT-6)-responsive T cells (28). The animal data have been supported by two clinical studies from Tsukaguchi et al. (4, 29). Furthermore, using a model of *in vitro* granulomas generated by DM2 patients' peripheral blood mononuclear cells infected with M. bovis BCG, the authors observed that PBMCs from GSH-supplemented patients produced enhanced levels of IFN-γ and controlled bacterial replication more efficiently than those from placebo-treated patients (30). In particular, the levels of IFN-γ were markedly reduced in samples from patients with poor glycemic control, which also failed to inhibit bacterial growth (31). Thus, the different immune responses involved in IFN-γ between TB with DM and without DM may be due not only to differences in the frequencies of innate and adaptive immune cells but also to uncontrolled hyperglycaemia.

Proinflammatory responses in the lungs can lead to tissue damage, disrupting normal tissue architecture and consequently compromising efficient gaseous exchange. An earlier study showed that progressive caseation of pulmonary granulomas did not occur in IFN-γ knockout mice with virulent *Mycobacterium avium* infection (32). However, mice treated with recombinant adenovirus IFN-γ showed high IFN-γ expression and exhibited significantly lower bacilli loads and pneumonia infected with H37Rv or the MDR strain (33). Notably, the reduction in IFN-γ impairs the phagocytic activity of the macrophage, thus altering the intracellular bacterial persistence and providing a replication niche with clinical consequences as cavitation develops (34). Through a comparison experiment with IFN-γ knockout and WT mice, Verma et al. showed that IFN-γ promoted the inflammatory cytokine storm response to cause lethal lung damages in a model of post-influenza methicillin-resistant *Staphylococcus aureus* pneumonia (35). Another study showed that administering IFN-γ after *P. aeruginosa* challenge resulted in a significant decrease in macroscopic lung pathology changes (36). Recent literature suggests that IFN-γ gene expression is positively correlated with skin lesion size in hamsters infected with *Leishmania braziliensis* (37). The data reported above indicate that the tissue damage from microbial infection, especially lung damage, is associated with IFN-γ.

Cell death is an essential attribute of tissue damage, including apoptosis, necroptosis, ferroptosis, and autophagy-dependent cell death. Lee et al. (38) reported that IFN-γ could attenuate necroptosis in collagen-induced arthritis mice by downregulating Th17 cell differentiation and inhibiting cellular FLICE-like inhibitory protein. *In vivo*, in mice bearing ovarian tumors, Wang et al. (39) reported that IFN-γ released from CD8+ T cells downregulated the expression of SLC3A2 and SLC7A11 and promoted tumor cell lipid peroxidation and ferroptosis. In addition, Orvedahl et al. (40) identified autophagy genes as central mediators of myeloid cell survival to IFN-γ mediated by TNF signaling via receptor interacting protein kinase 1 (RIPK1)- and caspase 8-mediated cell death. Therefore, the IFN-γ activity involved in various forms of cell death suggests that IFN-γ may improve a complex phenotype, such as “tissue damage,” by modulating cell death. IGRA detects IFN-γ released by specific T cells, reflecting the TB lymphocyte function and the host immune state to TB. Patients with severe TB, such as military TB and tuberculous meningitis, have been proven to have lower CD4 T-cell counts and impaired T-cell function. In our study, we retrospectively included 861 microbiological PTB participants for analysis. And the association between type 2 diabetes and pulmonary cavitation was found only in IGRA-positive TB patients. Our observation indicates that IFN-γ plays a crucial role in the development of cavitary with diabetes in TB patients.

The study had several limitations that should be considered. First, it was conducted in a specialized TB hospital with smear- or culture-positive confirmed TB patients, which resulted in a significantly lower number of negative IGRA patients (175) compared to positive IGRA patients (686). This retrospective study was based on the records of 5 years of clinical practice that were collected, indicating that further prospective cohort studies and follow-up studies are indispensable. Second, there were unexpected pathological changes in the enrolled TB patients, including the onset of infection, progression stages, and relapse. Addressing the phenomena between the different stages of M.tb infection and IFN-γ-related immunological regulation requires further evaluation with diabetic and latent TB animal models. Nevertheless, the population in the present study was an unbiased group of smear or culture-confirmed TB patients. Therefore, we believe that our results are represents more real-world outcomes.

In conclusion, in this study we found that there was a significant association between pulmonary cavitation and type 2 diabetes among the IGRA positive TB patients. This observation strongly suggests that different immunological mechanisms of pulmonary cavitary/DM would be employed in IGRA+ TB and IGRA- TB individuals. Further exploration of pulmonary cavitary/DM in IGRA+ and IGRA- TB patients would reveal more immunological characteristics of the tuberculosis disease.

## Data availability statement

The raw data supporting the conclusions of this article will be made available by the authors, without undue reservation.

## Ethics statement

The studies involving humans were approved by the Ethics Committee of the Third People's Hospital of Shenzhen (IRB No.: 2021-014-02). The studies were conducted in accordance with the local legislation and institutional requirements. The participants provided their written informed consent to participate in this study.

## Author contributions

Conceptualization and writing—review and editing: GZ, PZ, and GD. Formal analysis: PL and HL. Funding acquisition: GZ and GD. Investigation: MY and PZ. Methodology: XZ, PL, HL, and GZ. Writing—original draft: MY and PL. All authors have read and agreed to the published version of the manuscript.

## References

[1] WHO. Global tuberculosis report 2022. Geneva: World Health Organization (2022).

[2] Kumar Nathella P Babu S. Influence of diabetes mellitus on immunity to human tuberculosis. Immunology. (2017) 152:13–24. 10.1111/imm.12762 28543817 PMC5543489

[3] Zhan S Juan X Ren T Wang Y Fu L Deng G. Extensive radiological manifestation in patients with diabetes and pulmonary tuberculosis: a cross-sectional study. Ther Clin Risk Manag. (2022) 18:595–602. 10.2147/TCRM.S363328 35645562 PMC9137957

[4] Tsukaguchi K Okamura H Ikuno M Kobayashi A Fukuoka A Takenaka H. The relation between diabetes mellitus and IFN-gamma, IL-12 and IL-10 productions by CD4+ alpha beta T cells and monocytes in patients with pulmonary tuberculosis. Kekkaku. (1997) 72:617–22. 9423299

[5] Kumar NP George PJ Kumaran P Dolla CK Nutman TB Babu S. Diminished systemic and antigen-specific type 1, type 17, and other proinflammatory cytokines in diabetic and prediabetic individuals with latent *Mycobacterium tuberculosis* infection. J Infect Dis. (2014) 210:1670–8. 10.1093/infdis/jiu329 24907382 PMC4215076

[6] Cadena J Rathinavelu S Lopez-Alvarenga JC Restrepo BI. (2019). The re-emerging association between tuberculosis and diabetes: lessons from past centuries. Tuberculosis 116s:S89–s97. 10.1016/j.tube.2019.04.015 31085129 PMC6626679

[7] Dixon B. Diabetes and tuberculosis: an unhealthy partnership. Lancet Infect Dis. (2007) 7:444. 10.1016/S1473-3099(07)70144-5 17597568

[8] Badawi A Sayegh S Sallam M Sadoun E Al-Thani M Alam MW. The global relationship between the prevalence of diabetes mellitus and incidence of tuberculosis: 2000-2012. Glob J Health Sci. (2014) 7:183–91. 10.5539/gjhs.v7n2p183 25716376 PMC4796449

[9] Alemu A Bitew ZW Diriba G Gumi B. Co-occurrence of tuberculosis and diabetes mellitus, and associated risk factors, in Ethiopia: a systematic review and meta-analysis. IJID Regions. (2021) 1:82–91. 10.1016/j.ijregi.2021.10.004 35757829 PMC9216412

[10] Urbanowski ME Ordonez AA Ruiz-Bedoya CA Jain SK Bishai WR. Cavitary tuberculosis: the gateway of disease transmission. Lancet Infect Dis. (2020) 20:e117–28. 10.1016/S1473-3099(20)30148-1 32482293 PMC7357333

[11] Baker MA Harries AD Jeon CY Hart JE Kapur A Lönnroth K. The impact of diabetes on tuberculosis treatment outcomes: a systematic review. BMC Med. (2011) 9:81. 10.1186/1741-7015-9-81 21722362 PMC3155828

[12] Park SW Shin JW Kim JY Park IW Choi BW Choi JC. The effect of diabetic control status on the clinical features of pulmonary tuberculosis. Eur J Clin Microbiol Infect Dis. (2012) 31:1305–10. 10.1007/s10096-011-1443-3 22042559

[13] Bg T Bhakthavatchalam N Kk S. Clinicoradiological presentation of pulmonary tuberculosis in patients with diabetes mellitus at a tertiary care hospital. J Assoc Physicians India. (2022) 70:11–2.35443462

[14] Barreda NN Arriaga MB Aliaga JG Lopez K Sanabria OM Carmo TA. Severe pulmonary radiological manifestations are associated with a distinct biochemical profile in blood of tuberculosis patients with dysglycemia. BMC Infect Dis. (2020) 20:139. 10.1186/s12879-020-4843-0 32059707 PMC7023734

[15] Frydrych LM Bian G O'Lone DE Ward PA Delano MJ. Obesity and type 2 diabetes mellitus drive immune dysfunction, infection development, and sepsis mortality. J Leukoc Biol. (2018) 104:525–34. 10.1002/JLB.5VMR0118-021RR 30066958

[16] Flynn JL Chan J Triebold KJ Dalton DK Stewart TA Bloom BR. An essential role for interferon gamma in resistance to Mycobacterium tuberculosis infection. J Exp Med. (1993) 178:2249–54. 10.1084/jem.178.6.2249 7504064 PMC2191274

[17] Cooper AM Dalton DK Stewart TA Griffin JP Russell DG Orme IM. Disseminated tuberculosis in interferon gamma gene-disrupted mice. J Exp Med. (1993) 178:2243–7. 10.1084/jem.178.6.2243 8245795 PMC2191280

[18] Yang Y Wang H-J Hu W-L Bai G-N Hua C-Z. Diagnostic value of interferon-gamma release assays for tuberculosis in the immunocompromised population. Diagnostics. (2022) 12:453. 10.3390/diagnostics12020453 35204544 PMC8871457

[19] Blumberg HM Kempker RR. Interferon-γ release assays for the evaluation of tuberculosis infection. JAMA. (2014) 312:1460–1. 10.1001/jama.2014.4928 25291583 PMC4374979

[20] Chen X Yang Q Zhang M Graner M Zhu X Larmonier N. Diagnosis of active tuberculosis in China using an in-house gamma interferon enzyme-linked immunospot assay. Clin Vacc Immunol. (2009) 16:879–84. 10.1128/CVI.00044-09 19339489 PMC2691059

[21] WHO Guidelines Approved by the Guidelines Review Committee. Use of Glycated Haemoglobin (HbA1c) in the Diagnosis of Diabetes Mellitus: Abbreviated Report of a WHO Consultation. World Health Organization Copyright © Geneva: World Health Organization (2011). 26158184

[22] R.C. Team. R: A language and environment for statistical computing. In *R Foundation for Statistical Computing* (2022).

[23] Hosmer DW Lemeshow S. Model-building strategies and methods for logistic regression. In: Applied Logistic Regression. (2013), p. 89–151. 10.1002/9781118548387.ch4

[24] Sjoberg WK Curry DD Lavery M Larmarange JA. Reproducible summary tables with the summary package. R J. (2021) 13:570–80. 10.32614/RJ-2021-053

[25] Wickham H Averick M Bryan J Chang W D'Agostino L McGowan L. Welcome to the tidyverse. J Open Source Softw. (2019) 4:1689. 10.21105/joss.01686

[26] Bailey SL Grant P. 'The tubercular diabetic': the impact of diabetes mellitus on tuberculosis and its threat to global tuberculosis control. Clin Med. (2011) 11:344–7. 10.7861/clinmedicine.11-4-344 21853830 PMC5873743

[27] Cooper AM Khader SA. The role of cytokines in the initiation, expansion, and control of cellular immunity to tuberculosis. Immunol Rev. (2008) 226:191–204. 10.1111/j.1600-065X.2008.00702.x 19161425 PMC4298252

[28] Martens GW Arikan MC Lee J Ren F Greiner D Kornfeld H. Tuberculosis susceptibility of diabetic mice. Am J Respir Cell Mol Biol. (2007) 37:518–24. 10.1165/rcmb.2006-0478OC 17585110 PMC2048677

[29] Tsukaguchi K Okamura H Matsuzawa K Tamura M Miyazaki R Tamaki S. Longitudinal assessment of IFN-gamma production in patients with pulmonary tuberculosis complicated with diabetes mellitus. Kekkaku. (2002) 77:409–13. 12073618

[30] To K Cao R Yegiazaryan A Owens J Nguyen T Sasaninia K. Effects of oral liposomal glutathione in altering the immune responses against *Mycobacterium tuberculosis* and the *Mycobacterium bovis* BCG strain in individuals with type 2 diabetes. Front Cell Infect Microbiol. (2021) 11:657775. 10.3389/fcimb.2021.657775 34150674 PMC8211104

[31] Bobadilla-Del-Valle M Leal-Vega F Torres-Gonzalez P Ordaz-Vazquez A Garcia-Garcia ML Tovar-Vargas MLA. Mycobacterial growth inhibition assay (MGIA) as a host directed diagnostic tool for the evaluation of the immune response in subjects living with type 2 diabetes mellitus. Front Cell Infect Microbiol. (2021) 11:640707. 10.3389/fcimb.2021.640707 34084753 PMC8167894

[32] Ehlers S Benini J Held HD Roeck C Alber G Uhlig S. Alphabeta T cell receptor-positive cells and interferon-gamma, but not inducible nitric oxide synthase, are critical for granuloma necrosis in a mouse model of mycobacteria-induced pulmonary immunopathology. J Exp Med. (2001) 194:1847–59. 10.1084/jem.194.12.1847 11748285 PMC2193571

[33] Mata-Espinosa DA Mendoza-Rodriguez V Aguilar-Leon D Rosales R Lopez-Casillas F Hernandez-Pando R. Therapeutic effect of recombinant adenovirus encoding interferon-gamma in a murine model of progressive pulmonary tuberculosis. Molec Ther. (2008) 16:1065–72. 10.1038/mt.2008.69 18431363

[34] Ayelign B Negash M Genetu M Wondmagegn T Shibabaw T. Immunological impacts of diabetes on the susceptibility of *Mycobacterium tuberculosis*. J Immunol Res. (2019) 2019:6196532. 10.1155/2019/6196532 31583258 PMC6754884

[35] Verma AK Bauer C Palani S Metzger DW Sun K. IFN-gamma drives TNF-alpha hyperproduction and lethal lung inflammation during antibiotic treatment of postinfluenza staphylococcus aureus pneumonia. J Immunol. (2021) 207:1371–6. 10.4049/jimmunol.2100328 34380647

[36] Johansen HK Hougen HP Rygaard J Hoiby N. Interferon-gamma (IFN-gamma) treatment decreases the inflammatory response in chronic *Pseudomonas aeruginosa* pneumonia in rats. Clin Exp Immunol. (1996) 103:212–8. 10.1046/j.1365-2249.1996.d01-618.x 8565302 PMC2200342

[37] Paiva MB Ribeiro-Romão RP Resende-Vieira L Braga-Gomes T Oliveira MP Saavedra AF. A cytokine network balance influences the fate of leishmania (viannia) braziliensis infection in a cutaneous leishmaniasis hamster model. Front Immunol. (2021) 12:656919. 10.3389/fimmu.2021.656919 34276650 PMC8281932

[38] Prada-Medina CA Fukutani KF Pavan Kumar N Gil-Santana L Babu S Lichtenstein F. Systems immunology of diabetes-tuberculosis comorbidity reveals signatures of disease complications. Sci Rep. (2017) 7:1999. 10.1038/s41598-017-01767-4 28515464 PMC5435727

[39] Wang WM Green M Choi JE Gijon M Kennedy PD Johnson JK. CD8(+) T cells regulate tumour ferroptosis during cancer immunotherapy. Nature. (2019) 569:270. 10.1038/s41586-019-1170-y 31043744 PMC6533917

[40] Orvedahl A McAllaster MR Sansone A Dunlap BF Desai C Wang YT. Autophagy genes in myeloid cells counteract IFN gamma-induced TNF-mediated cell death and fatal TNF-induced shock. Proc Natl Acad Sci U S A. (2019) 116:16497–506. 10.1073/pnas.1822157116 31346084 PMC6697821

