# Supplementary material for: The association between type 2 diabetes and pulmonary cavitation revealed among IGRA-positive tuberculosis patients
Source: Front Med (Lausanne). 2023 Dec 6;10:1245316. doi: 10.3389/fmed.2023.1245316 (PMC10731020; doi:10.3389/fmed.2023.1245316)
Supplement: Supplementary file 1 [file Data_Sheet_1.PDF]

Supplementary Material

The Association between Type 2 Diabetes and Pulmonary Cavitation Revealed among IGRA-Positive Tuberculosis Patients

Min Yang[1#]; Pei Li[1#]; Han Liu[1]; Xiaojie Zhu[2]; Guofeng Zhu[1]; Peize Zhang[1*]; Guofang Deng[1*]

[#] **These authors contributed equally to this work.**

*** Correspondence:**
Peize Zhang, MD/PhD, Email: 82880246@qq.com;

Guofang Deng, MD, Email: jxxk1035@yeah.net;

1 Supplementary Tables

eTable 1. Demographic and clinical characteristics of patients with pulmonary tuberculosis in the second department of pulmonary medicine and tuberculosis from 2014 to 2019 (n=861)

**Characteristic**	**Total (861)**
**Socio-demographic**	
Age, years, mean (SD)	36.7 (13)
Gender, n (%)	
Male	548 (63.6)
Female	313 (36.3)

Smoke status, n (%)	
Never	601 (69.8)
Current	208 (24.2)
Former	52 (6.0)
Drink status, n (%)	
Never	734 (85.2)
Current	116 (13.5)
Former	11 (1.3)
Marital status, n (%)	
Married	550 (63.9)
Single	311 (36.1)
Work status, n (%)	
Employed	591 (68.6)
Unemployed	270 (31.4)

Ethnic groups, n (%)	
Han	835 (97.0)
Others	26 (3.0)
**Clinical Characteristics**	
IGRA, n (%)	
IGRA (+)	686 (79.7)
IGRA (-)	175 (20.3)
Pulmonary cavitation, n (%)	
Yes	351 (40.8)
No	510 (59.2)
Diabetes mellitus (DM), n (%)	
DM (+)	119（13.8）
DM (-)	742（86.2）
Blood biochemical indexes	

WBC (10^9/L), mean (SD)	7.7 (3.0)
GRA (10^9/L), mean (SD)	5.5 (3.4)
LYN (10^9/L), mean (SD)	1.4 (0.6)
MONO (10^9/L), mean (SD)	0.7 (0.6)
HGB (g/L), mean (SD)	123.0 (20.6)

eTable 2a. Univariate and multivariate logistic regression analyses of risk factors of IGRA in cavitary TB patients (n=351)

**Characteristic**	**Univariate analyses**				**Multivariate analyses**	
	**IGRA (-) (n=68)**	**IGRA (+) (n=283)**	**Unadjusted OR (95% CI)**	**P values**	**Adjusted OR (95% CI)**	**P values**
Age, years, mean (SD)	38.9 (13.5)	37.9 (13.3)	0.99 (0.97，1.01)	0.553		
Gender, n (%)						
Female	19 (27.9)	69 (24.4)	Reference			
Male	49 (72.1)	214 (75.6)	0.83 (0.46，1.54)	0.543		
Smokers, n (%)						
Never	42 (61.8)	159 (56.2)	Reference			

Current	21 (30.9)	100 (35.3)	1.26 (0.71，2.28)	0.439
Former	5 (7.4)	24 (8.5)	1.27 (0.49，3.94)	0.649
Drinkers, n (%)				
Never	56 (82.4)	216 (76.3)	Reference	
Current	11 (16.2)	64 (22.6)	1.51(0.77，3.19)	0.252
Former	1 (1.5)	3 (1.1)	0.78 (0.1，15.89)	0.829
Work status, n (%)				
Unemployed	44 (64.7)	177 (62.5)	Reference	
Employed	24 (35.3)	106 (37.5)	1.10 (0.64，1.93)	0.74
Marital status, n (%)				

Single	23 (33.8)	97 (34.3)	Reference			
Married	45 (66.2)	186 (65.7)	0.98 (0.55，1.70)	0.944		
Ethnic groups, n (%)						
Han	66 (97.1)	268 (94.7)	Reference			
Others	2 (2.9)	15 (5.3)	1.85 (0.50，11.90)	0.423		
Blood biochemical indexes						
WBC (10^9/L)	8.2 (2.6)	8.4 (3.3)	1.02 (0.94，1.12)	0.588		
GRA (10^9/L)	6.13 (2.4)	6.06 (3.1)	0.99 (0.91，1.09)	0.861		
LYN (10^9/L)	1.24 (0.6)	1.43 (0.6)	1.74 (1.1，2.84)	0.021	1.53 (0.93，2.59)	0.102
MONO (10^9/L)	0.65 (0.3)	0.73 (0.3)	2.16 (0.93，5.39)	0.083		

HGB (g/L)	117.51 (21.9)	123.36 (20.1)	1.01 (1.00，1.03)	0.037	1.01 (0.99，1.02)	0.213
Diabetes mellitus (DM), n(%)						
DM (+)	56 (82.4)	217(76.7)	1.42 (0.74，2.92)	0.314		
DM (-)	12 (17.6)	66 (23.3)	Reference			

[1]n (%); Mean (SD); Reference used as control for comparison

[2]OR = Odds Ratio, CI = Confidence Interval

WBC, white blood cell; GRA, neutrophilic granulocyte; LYN, lymphocyte; MONO, monocyte; HGB, haemoglobulin; ALB, albumin; CRP, C-reactive protein, significant differences ($p < 0.05$) are bolded.

eTable 2b. Univariate and multivariate logistic regression analyses of risk factors of type 2 diabetes in cavitary TB patients (n=351)

Characteristic	Univariate analyses				Multivariate analyses	
	**Diabetes, (n=78)**	**Non- diabetes, (n=273)**	**Unadjusted OR (95% CI)**	**P values**	**Adjusted OR (95% CI)**	**P values**
Age, years, mean (SD)	48.8 (8.9)	35.0(12.8)	1.09 (1.07, 1.12)	<0.001	1.08 (1.05, 1.11)	**<0.001**
Gender, n (%)						
Female	13 (16.7%)	198 (72.5%)	Reference			
Male	65(83.3%)	75(27.5%)	0.53 (0.26, 0.98)	0.055		
Smokers, n (%)						
Never	32(41%)	169(61.9%)	Reference			
Current	36(46.2%)	85(31.1%)	2.24 (1.30, 3.86)	0.004		
Former	10(12.8%)	19(7%)	2.78 (1.15, 6.44)	0.019		
Drinkers, n (%)						
Never	56(71.8%)	216(79.1%)	Reference			
Current	21(26.9%)	54(19.8%)	1.50 (0.82, 2.66)	0.173		
Former	1(1.3%)	3(1.1%)	1.28 (0.06, 10.26)	0.829		

Work status, n (%)						
Unemployed	47(60.3%)	174(63.7%)	Reference			
Employed	31(39.7%)	99(36.3%)	1.16 (0.69, 1.94)	0.575		
Marital status, n (%)						
Single	3(3.8%)	117(42.9%)	Reference		Reference	
Married	75(96.2%)	156(57.1%)	18.75 (6.78, 77.82)	<0.001	6.18 (2.03, 26.86)	**0.004**
Ethnic groups, n (%)						
Han	77(98.7%)	257(94.1%)	Reference			
Others	1(1.3%)	16(5.9%)	0.21 (0.01, 1.05)	0.131		
Blood biochemical indexes						
WBC (10^9/L)	8.6 (2.9)	8.3 (3.3)	1.03 (0.95, 1.11)	0.455		
GRA (10^9/L)	6.2 (2.6)	6.0 (3.0)	1.02 (0.93, 1.10)	0.688		
LYN (10^9/L)	1.5 (0.6)	1.4 (0.6)	1.43 (0.95, 2.16)	<0.001		
MONO (10^9/L)	0.8 (0.3)	0.7 (0.3)	1.51 (0.72, 3.09)	0.263		
HGB (g/L)	126.3 (20.3)	121.1 (20.5)	1.01 (1.00，1.03)	0.047	1.02 (1.00, 1.03)	**0.019**
IGRA						

Negative	12(15.4%)	56(20.5%)	1.42 (0.74, 2.92)	0.314
Positive	66(84.6%)	217(79.5%)	Reference	

[1]n (%); Mean (SD); Reference used as control for comparison

[2]OR = Odds Ratio, CI = Confidence Interval

WBC, white blood cell; GRA, neutrophilic granulocyte; LYN, lymphocyte; MONO, monocyte; HGB, haemoglobulin; ALB, albumin; CRP, C-reactive protein, significant differences ($p < 0.05$) are bolded.

eTable 3a. Univariate and multivariate logistic regression analyses of risk factors of IGRA in non-cavitary TB patients (n=510)

	**Univariate analyses**				**Multivariate analyses**	
**Characteristic**	**IGRA (-) (n=107)**	**IGRA (+) (n=403)**	**Unadjusted OR (95% CI)**	**P values**	**Adjusted OR (95% CI)**	**P values**
Age, years, mean (SD)	39.7 (14.1)	34.7(12.1)	0.97 (0.95, 0.99)	0.000303	0.97(0.96, 0.99)	**0.0008**
Gender, n (%)						
Female	46 (43)	179 (44.4)	Reference			
Male	61(57)	224(55.6)	1.06 (0.69, 1.63)	0.792		
Smokers, n (%)						
Never	81(75.7)	319(79.2)	Reference			

Current	19(17.8)	68(16.9)	0.91 (0.53, 1.63)	0.740
Former	7(6.5)	16(4)	0.58 (0.24, 1.55)	0.247
Drinkers, n (%)				
Never	95(88.8)	367(91.1)	Reference	
Current	9(8.4)	32(7.9)	0.92 (0.44, 2.11)	0.833
Former	3(2.8)	4(1)	0.35 (0.07, 1.78)	0.168
Work status, n (%)				
Unemployed	78(72.9)	292(72.5)	Reference	
Employed	29(27.1)	111(27.5)	1.02 (0.64, 1.67)	0.928
Marital status, n (%)				

Single	30(28)	161(40)	Reference			
Married	77(72)	242(60)	0.59(0.36, 0.93)	0.025		
Ethnic groups, n (%)						
Han	104(97.2)	397(98.5)	Reference			
Others	3(2.8)	6(1.5)	0.52 (0.14, 2.52)	0.366		
Blood biochemical indexes						
WBC (10^9/L)	7.93 (3.6)	7.04 (2.4)	0.9 (0.83, 0.96)	0.003	0.9 (0.84, 0.97)	**0.0077**
GRA (10^9/L)	5.78 (3.5)	4.92 (3.7)	0.95 (0.88, 1.0)	0.079		
LYN (10^9/L)	1.32 (0.7)	1.42 (0.6)	1.28 (0.91, 1.85)	0.177		
MONO (10^9/L)	0.64 (0.3)	0.71 (0.8)	1.21 (0.85, 2.33)	0.473		

HGB (g/L)	119.52 (22.0)	124.49 (20.1)	1.01 (1.00, 1.02)	0.028	1.01 (1.0, 1.02)	**0.0496**
Diabetes mellitus (DM), n(%)						
DM (+)	94(87.9)	375(93.1)	0.54 (0.27, 1.11)	0.0824		
DM (-)	13(12.1)	28(6.9)	Reference			

[1]n (%); Mean (SD); Reference used as control for comparison

[2]OR = Odds Ratio, CI = Confidence Interval

WBC, white blood cell; GRA, neutrophilic granulocyte; LYN, lymphocyte; MONO, monocyte; HGB, haemoglobulin; ALB, albumin; CRP, C-reactive protein, significant differences ($p < 0.05$) are bolded.

eTable 3b. Univariate and multivariate logistic regression analyses of risk factors of type 2 diabetes in non-cavitary TB patients (n=510)

	**Univariate analyses**				**Multivariate analyses**	
**Characteristic**	**Diabetes, (n=41)**	**Non- diabetes, (n=469)**	**Unadjusted OR (95% CI)**	**P values**	**Adjusted OR (95% CI)**	**P values**
Age, years, mean (SD)	51.2 (9.6)	34.4 (12.0)	1.11(1.08, 1.14)	<0.001	1.10(1.07, 1.14)	**<0.001**
Gender, n (%)						
Female	8 (19.5%)	217 (46.3%)	Reference		Reference	
Male	33 (80.5%)	252 (53.7%)	0.28(0.12, 0.59)	0.002	0.38(0.15, 0.86)	**0.027**
Smokers, n (%)						
Never	24 (58.5%)	376 (80.2%)	Reference			
Current	12 (29.3%)	75 (16.0%)	2.51 (1.16, 5.14)	0.014		

Former	5 (12.2%)	18 (3.8%)	4.35 (1.35, 12.03)	0.007
Drinkers, n (%)				
Never	32 (78.0%)	430 (91.7%)	Reference	
Current	6 (14.6%)	35 (7.5%)	2.30 (0.82, 5.54)	0.081
Former	3 (7.3%)	4 (0.9%)	10.08 (1.92, 47.63)	0.003
Work status, n (%)				
Unemployed	30 (73.2%)	340 (72.5%)	Reference	
Employed	11 (26.8%)	129 (27.5%)	0.97 (0.45, 1.93)	0.926
Marital status, n (%)				
Single	4 (9.8%)	187 (39.9%)	Reference	
Married	37 (90.2%)	282 (60.1%)	6.13(2.41, 20.74)	<0.001

Ethnic groups, n (%)						
Han	40 (97.6%)	461 (98.3%)	Reference			
Others	1 (2.4%)	8 (1.7%)	1.44 (0.08, 8.14)	0.734		
Blood biochemical indexes						
WBC (10^9/L)	8.6 (3.5)	7.1 (2.6)	1.16 (1.06, 1.28)	0.001	1.12 (1.00, 1.25)	0.052
GRA (10^9/L)	6.4 (3.6)	5.0 (3.7)	1.06 (0.99, 1.14)	0.075		
LYN (10^9/L)	1.3 (0.5)	1.4 (0.7)	0.84 (0.48, 1.38)	0.530		
MONO (10^9/L)	0.7 (0.3)	0.7 (0.8)	1.01 (0.50, 1.37)	0.967		
HGB (g/L)	124.3 (18.6)	123.4 (20.7)	1.00 (0.99, 1.02)	0.782		
IGRA						
Negative	13 (31.7%)	94(20%)	0.54 (0.27, 1.11)	0.082		

Positive	28 (68.3%)	375 (80%)	Reference

[1]n (%); Mean (SD); Reference used as control for comparison

[2]OR = Odds Ratio, CI = Confidence Interval

WBC, white blood cell; GRA, neutrophilic granulocyte; LYN, lymphocyte; MONO, monocyte; HGB, haemoglobulin; ALB, albumin; CRP, C-reactive protein, significant differences ($p < 0.05$) are bolded.

eTable 4a. Univariate and multivariate logistic regression analyses of risk factors of IGRA in TB patients with type 2 diabetes (n=119)

**Characteristic**	**Univariate analyses**				**Multivariate analyses**	
	**IGRA (-) (n=25)**	**IGRA (+) (n=94)**	**Unadjusted OR (95% CI)**	**P values**	**Adjusted OR (95% CI)**	**P values**
Age, years, mean (SD)	50.2 (8.4)	49.5 (9.4)	0.99(0.94, 1.04)	0.734		
Gender, n (%)						
Female	7 (28)	14 (14.9)	Reference			
Male	18 (72)	80 (85.1)	0.45(0.16, 1.33)	0.133		
Smokers, n (%)						
Never	13 (52)	43 (45.7)	Reference			

Current	9 (36)	39 (41.5)	1.31 (0.51, 3.49)	0.579		
Former	3 (12)	12 (12.8)	1.21 (0.32, 5.89）	0.792		
Drinkers, n (%)						
Never	21 (84)	67 (71.3)	Reference			
Current	4 (16)	23 (24.5)	1.802239e+00 (0.76, 2.13)	0.324		
Former	0	4(4.3)	4.905650e+06（1.090636e-53, NA）	0.990		
Work status, n (%)						
Unemployed	19 (76)	58(61.7)	Reference			
Employed	6 (24)	36(38.3)	1.97 (0.75, 5.81)	0.189	2.22(0.78, 7.21)	0.155
Marital status, n (%)						

Single	1 (4)	6 (6.4)	Reference			
Married	24 (96)	88 (93.6)	0.61 (0.03, 3.82)	0.6557		
Ethnic groups, n (%)						
Han	25 (100)	92 (97.9)	Reference			
Others	0	2(2.1)	1564623.06 (1.163107e-64, NA)	0.0146		
Cavitary, n (%)						
Cavitary	13 (52)	28 (29.8)	2.55 (1.04, 6.37)	0.0413	2.10 (0.80, 5.52)	0.129
Non-cavitary	12 (48)	66 (70.2)	Reference			
Blood biochemical indexes						
WBC (10^9/L)	9.1 (3.7)	8.5 (2.9)	0.94 (0.82, 1.08)	0.372		

GRA (10^9/L)	7.1 (3.7)	6 (2.7)	0.89 (0.77, 1.02)	0.0991	0.88 (0.73, 1.04)	0.134
LYN (10^9/L)	1.2 (0.6)	1.5 (0.6)	3.18 (1.29, 9.07)	0.0196	2.36 (0.87, 7.42)	0.113
MONO (10^9/L)	0.6 (0.3)	0.8 (0.3)	3.33 (0.76, 18.85)	0.140	4.8 (0.81, 37.99)	0.106
HGB (g/L)	119.4 (20.9)	127.3 (19.1)	1.02 (1.00, 1.04)	0.0799		

[1]n (%); Mean (SD); Reference used as control for comparison

[2]OR = Odds Ratio, CI = Confidence Interval

WBC, white blood cell; GRA, neutrophilic granulocyte; LYN, lymphocyte; MONO, monocyte; HGB, haemoglobulin; ALB, albumin; CRP, C-reactive protein, significant differences ($p < 0.05$) are bolded.

eTable 4b. Univariate and multivariate logistic regression analyses of risk factors of cavitary in TB patients with type 2 diabetes (n=119)

**Characteristic**	**Univariate analyses**				**Multivariate analyses**	
	**Cavitary, (n=41)**	**Noncavitary, (n=78)**	**Unadjusted OR (95% CI)**	**P values**	**Adjusted OR (95% CI)**	**P values**
Age, years, mean (SD)	51.2 (9.6)	48.8 (8.9)	1.00 (0.99, 1.01)	0.498		
Gender, n (%)						
Female	8 (19.5%)	65 (83.3%)	Reference			
Male	33 (80.5%)	13 (16.7%)	0.44(0.32, 0.60)	<0.001	0.54(0.38, 0.77)	**<0.001**
Smokers, n (%)						
Never	24 (58.5%)	32 (41%)	Reference			
Current	12 (29.3%)	36 (46.2%)	2.52 (1.76, 3.62)	<0.001		

Former	5 (12.2%)	10 (12.8%)	2.35 (1.20, 4.62）	0.013		
Drinkers, n (%)						
Never	32 (78%)	56 (71.8%)	Reference			
Current	6 (14.6%)	21 (26.9%)	3.07 (1.96, 4.88)	<0.001	2.26 (1.39, 3.70)	**0.001**
Former	3(7.3%)	1(1.3%)	1.49（0.29, 6.83）	0.602	0.70 (0.13, 3.46)	0.661
Work status, n (%)						
Unemployed	30 (73.2%)	47(60.3%)	Reference			
Employed	11 (26.8%)	31(39.7%)	1.50 (1.09, 2.06)	0.013	1.61 (1.14, 2.26)	**0.006**
Marital status, n (%)						
Single	4 (9.8%)	3 (3.8%)	Reference			
Married	37 (90.2%)	75 (96.2%)	0.88 (0.65, 1.20)	0.425		

Ethnic groups, n (%)						
Han	40 (97.6%)	77 (98.7%)	Reference			
Others	1(2.4%)	1(1.3%)	3.59 (1.56, 8.95)	0.004	3.50 (1.46, 9.00)	**0.006**
IGRA						
Negative	13 (31.7%)	12 (15.4%)	Reference			
Positive	28 (68.3%)	66 (84.6%)	2.55 (1.04, 6.37)	0.0413	2.1 (0.80, 5.52)	0.129
Blood biochemical indexes						
WBC (10^9/L)	8.6 (3.5)	8.6 (2.9)	1.15 (1.09, 1.22)	<0.001	1.12 (1.06, 1.19)	**<0.001**
GRA (10^9/L)	6.4 (3.6)	6.2 (2.6)	1.12 (1.06, 1.19)	<0.001		
LYN (10^9/L)	1.3 (0.5)	1.5 (0.6)	0.91 (0.71, 1.14)	0.417		
MONO (10^9/L)	0.7(0.3)	0.8(0.3)	1.02 (0.79, 1.30)	0.847		

HGB (g/L)	124.3 (18.6)	126.3 (0.3)	0.99 (0.99, 1.00)	0.143

[1]n (%); Mean (SD); Reference used as control for comparison

[2]OR = Odds Ratio, CI = Confidence Interval

WBC, white blood cell; GRA, neutrophilic granulocyte; LYN, lymphocyte; MONO, monocyte; HGB, haemoglobulin; ALB, albumin; CRP, C-reactive protein, significant differences ($p < 0.05$) are bolded.

eTable 5a. Univariate and multivariate logistic regression analyses of risk factors of IGRA in TB patients without type 2 diabetes (n=742)

Characteristic	Univariate analyses				Multivariate analyses	
	**IGRA (-) (n=150)**	**IGRA (+) (n=592)**	**Unadjusted OR (95% CI)**	**P values**	**Adjusted OR (95% CI)**	**P values**
Age, years, mean (SD)	37.6 (13.8)	33.8 (11.8)	0.98(0.96, 0.99)	0.000873	0.98 (0.96, 0.99)	**0.001**
Gender, n (%)						
Female	58 (38.7)	234 (39.5)	Reference			
Male	92 (61.3)	358 (60.5)	1.04 (0.72, 1.5)	0.847		
Smokers, n (%)						
Never	110((73.3)	435 (73.5)	Reference			
Current	31(20.7)	129 (21.8)	1.05 (0.68, 1.66)	0.822		

Former	9(6)	28(4.7)	0.79 (0.37, 1.81)	0.546
Drinkers, n (%)				
Never	130 (86.7)	516 (87.2)	Reference	
Current	16 (10.7)	73 (12.3)	1.15 (0.66, 2.11)	0.634
Former	4 (2.7)	3 (0.5)	0.19 (0.04, 0.87)	0.031
Work status, n (%)				
Unemployed	103 (68.7)	411 (69.4)	Reference	
Employed	47 (31.3)	181 (30.6)	0.97 (0.66, 1.43)	0.857
Marital status, n (%)				
Single	52 (34.7)	252 (42.6)	Reference	
Married	98 (65.3)	340 (57.4)	0.72 (0.49, 1.04)	0.08

Ethnic groups, n (%)						
Han	145 (96.7)	573 (96.8)	Reference			
Others	5 (3.3)	19 (3.2)	0.96 (0.38, 2.94)	0.939		
Cavitary, n (%)						
Cavitary	94 (62.7)	375 (63.3)	0.97 (0.67, 1.41)	0.878		
Non-cavitary	56 (37.3)	217 (36.7)	Reference			
Blood biochemical indexes						
WBC (10^9/L)	7.8 (3.1)	7.5 (2.8)	0.96(0.90, 1.02)	0.156	0.96 (0.90, 1.02)	0.150
GRA (10^9/L)	5.7 (2.9)	5.3 (3.6)	0.97 (0.92, 1.02)	0.21		
LYN (10^9/L)	1.3 (0.7)	1.4 (0.6)	1.31 (0.98, 1.79)	0.078		
MONO (10^9/L)	0.6 (0.3)	0.7 (0.7)	1.34 (0.90, 2.41)	0.281		

HGB (g/L)	118.6 (22.2)	123.5 (20.2)	1.01 (1.00, 1.02)	0.011	**1.01** (1.00, 1.02)	**0.017**

[1]n (%); Mean (SD); Reference used as control for comparison

[2]OR = Odds Ratio, CI = Confidence Interval

WBC, white blood cell; GRA, neutrophilic granulocyte; LYN, lymphocyte; MONO, monocyte; HGB, haemoglobulin; ALB, albumin; CRP, C-reactive protein, significant differences ($p < 0.05$) are bolded.

eTable 5b. Univariate and multivariate logistic regression analyses of risk factors of cavitary in TB patients without type 2 diabetes (n=742)

Characteristic	Univariate analyses				Multivariate analyses	
	**Cavitary, (n=469)**	**Noncavitary, (n=273)**	**Unadjusted OR (95% CI)**	**P values**	**Adjusted OR (95% CI)**	**P values**
Age, years, mean (SD)	34.4 (12.0)	35.0 (12.8)	0.97 (0.93, 1.01)	0.167		
Gender, n (%)						
Female	217 (46.3%)	198 (72.5%)	Reference			
Male	252 (53.7%)	75 (27.5%)	0.83 (0.32, 2.27)	0.699		
Smokers, n (%)						
Never	376((80.2%)	169 (61.9%)	Reference			
Current	75(16.0%)	85 (31.1%)	2.25 (0.98, 5.34)	0.059		

Former	18(3.8%)	19(7.0%)	1.50 (0.47, 5.35)	0.507
Drinkers, n (%)				
Never	430 (91.7%)	216 (79.1%)	Reference	
Current	35 (7.5%)	54 (19.8%)	2.00 (0.77, 5.91)	0.177
Former	4 (0.9%)	3 (1.1%)	0.19 (0.01, 1.56)	0.158
Work status, n (%)				
Unemployed	340 (72.5%)	174 (63.7%)	Reference	
Employed	129 (27.5%)	99 (36.3%)	1.80 (0.80, 4.23)	0.163
Marital status, n (%)				
Single	187 (39.9%)	117 (42.9%)	Reference	
Married	282 (60.1%)	156 (57.1%)	2.70 (0.57, 14.32)	0.208

Ethnic groups, n (%)				
Han	461 (98.3%)	257 (94.1%)	Reference	
Others	8 (1.7%)	16 (5.9%)	0.52 (0.02, 13.37)	0.646
IGRA				
Negative	94 (20.0%)	56 (20.5%)	Reference	
Positive	375 (80.0%)	217 (79.5%)	0.97 (0.67, 1.41)	0.878
Blood biochemical indexes				
WBC (10^9/L)	7.1 (2.6)	8.3 (3.3)	1.00 (0.89, 1.13)	0.995
GRA (10^9/L)	5.0 (3.7)	6.0 (3.0)	0.98 (0.86, 1.12)	0.747
LYN (10^9/L)	1.4 (0.7)	1.4 (0.6)	1.67 (0.85, 3.51)	0.153
MONO (10^9/L)	0.7 (0.8)	0.7 (0.3)	1.68 (0.52, 6.01)	0.401

HGB (g/L)	123.4 (20.7)	121.1 (20.5)	1.01 (0.98, 1.03)	0.590

[1]n (%); Mean (SD); Reference used as control for comparison

[2]OR = Odds Ratio, CI = Confidence Interval

WBC, white blood cell; GRA, neutrophilic granulocyte; LYN, lymphocyte; MONO, monocyte; HGB, haemoglobulin; ALB, albumin; CRP, C-reactive protein, significant differences ($p < 0.05$) are bolded.

2 Supplementary Figures and Figure Legends

eFigure 1. CT chest showing cavities and multi-nodules.

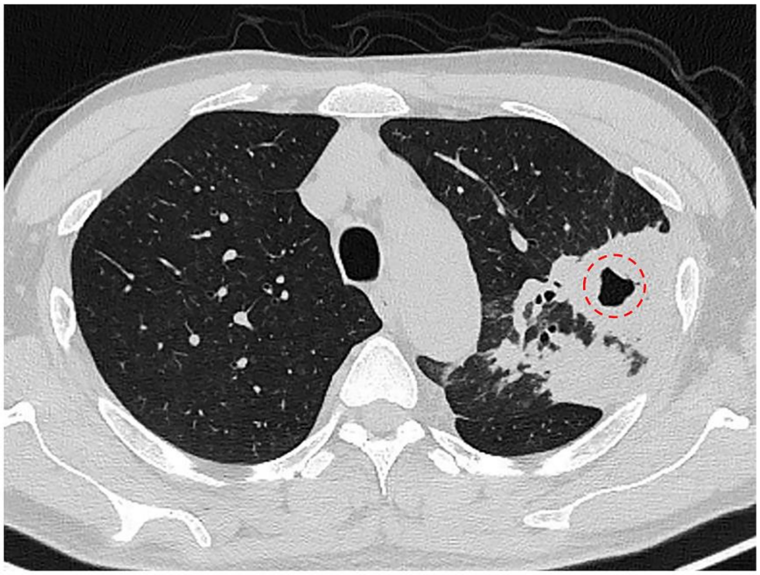

Caseous consolidation with cavity (red circle) in a TB patient with diabetes

frontiers
